# Gender Difference in Ventricular Response to Aortic Stenosis: Insight from Cardiovascular Magnetic Resonance

**DOI:** 10.1371/journal.pone.0121684

**Published:** 2015-03-26

**Authors:** Joo Myung Lee, Sung-Ji Park, Seung-Pyo Lee, Eunah Park, Sung-A Chang, Hyung-Kwan Kim, Whal Lee, Yong-Jin Kim, Sang-Chol Lee, Seung Woo Park, Dae-Won Sohn, Yeon Hyeon Choe

**Affiliations:** 1 Cardiovascular Center, Sungkyunkwan University School of Medicine, Seoul, Korea; 2 Department of Internal Medicine, Sungkyunkwan University School of Medicine, Seoul, Korea; 3 Department of Radiology, Seoul National University Hospital, Sungkyunkwan University School of Medicine, Seoul, Korea; 4 Cardiovascular Imaging Center, Samsung Medical Center, Sungkyunkwan University School of Medicine, Seoul, Korea; Sapienza University of Rome, ITALY

## Abstract

**Background:**

Although left ventricular hypertrophy (LVH) and remodeling is associated with cardiac mortality and morbidity, little is known about the impact of gender on the ventricular response in aortic stenosis (AS) patients. This study aimed to analyze the differential effect of gender on ventricular remodeling in moderate to severe AS patients.

**Methods and Results:**

A total of 118 consecutive patients (67±9 years; 63 males) with moderate or severe AS (severe 81.4%) underwent transthoracic echocardiography and cardiovascular magnetic resonance (CMR) within a 1-month period in this two-center prospective registry. The pattern of LV remodeling was assessed using the LV mass index (LVMI) and LV remodeling index (LVRI; LV mass/LV end-diastolic volume) by CMR. Although there were no differences in AS severity parameters nor baseline characteristics between genders, males showed a significantly higher LVMI (102.6±29.1g/m^2^ vs. 86.1±29.2g/m^2^, p=0.003) and LVRI (1.1±0.2 vs. 1.0±0.3, p=0.018), regardless of AS severity. The LVMI was significantly associated with aortic valve area (AVA) index and valvuloarterial impedance in females, whereas it was not in males, resulting in significant interaction between genders (PInteraction=0.007/0.014 for AVA index/valvuloarterial impedance, respectively). Similarly, the LVRI also showed a significantly different association between male and female subjects with the change in AS severity parameters (PInteraction=0.033/<0.001/0.029 for AVA index/transaortic mean pressure gradient/valvuloarterial impedance, respectively).

**Conclusion:**

Males are associated with greater degree of LVH and higher LVRI compared to females at moderate to severe AS. However, females showed a more exaggerated LV remodeling response, with increased severity of AS and hemodynamic loads, than males.

## Introduction

Chronic left ventricular (LV) pressure overload caused by aortic stenosis (AS) typically leads to a hypertrophic response of the myocardium, which might be an adaptive response of the myocardium in an effort to normalize increased wall tension and to maintain cardiac output [1]. However, this cascade of compensatory responses alters the myocardium, leading to adverse consequences such as ischemia, diastolic dysfunction and impairment of systolic function and heart failure in the long term [2]. Accordingly, accurate assessment of left ventricular hypertrophy (LVH) plays an important role in assessing prognosis in AS patients.

Despite some past evidence of gender-specific differences in these ventricular responses to the chronic pressure overload [3], controversy still surrounds this issue and its impact on clinical outcomes, especially regarding its role following surgical aortic valve replacement (SAVR) or transcatheter aortic valve replacement (TAVR)[4–8]. Furthermore, the results of these reports have been mixed and consequently, confusing to interpret.

Previous studies examining gender-specific differences of the ventricular response to AS were undertaken only with transthoracic echocardiography (TTE) [9, 10], which may have several limitations when compared to cardiovascular magnetic resonance (CMR). CMR offers more precise measurements of LV mass, volume and wall thickness than does TTE [11]. Therefore, accurate assessment of the myocardial response to chronic pressure overload using CMR might provide a novel and accurate mechanistic explanation for gender-specific differences in clinical outcomes in patients with significant AS. Therefore, we investigated whether gender-specific differences in ventricular hypertrophy and remodeling would be evident in response to moderate or severe AS with the use of CMR.

## Methods

This study was conducted according to the principles outlined in the Declaration of Helsinki. All patients gave written informed consent to this prospective study, the protocol of which was approved by the ethical committee and the Institutional Review Board of both institutions (Seoul National University Hospital and Samsung Medical Center).

### Patient population

A total of 118 patients with moderate or severe AS, i.e. maximal transaortic velocity >3m/sec or transaortic mean pressure gradient (PG) >30mmHg and aortic valve area ≤1.5cm^2^ with normal LV systolic function (EF>40%), were consecutively enrolled to this prospective two-center registry (76 patients from Seoul National University Hospital and 42 patients from Samsung Medical Center). All patients diagnosed with moderate or severe AS by echocardiography were enrolled. For research purposes, from June 2008, the participating centers prospectively collected CMR data of the patients with moderate to severe AS. In order to explore, using CMR, the isolated effect of AS on ventricular remodeling, our cohort was carefully selected to avoid patients with confounding drivers of LV remodeling. Therefore, the exclusion criteria for this cohort (and for the CMR evaluation) were: patients with prior myocardial infarction; significant valvular disease (moderate or severe mitral, tricuspid or pulmonic valve disease and moderate to severe aortic regurgitation) other than AS; a clinical diagnosis of co-existing cardiomyopathy including hypertrophic cardiomyopathy, amyloidosis, uncontrolled hypertension (>180/120mmHg); severe renal failure (estimated glomerular filtration rate <30mL/min/kg); and disseminated malignancy. Those patients with moderate or severe AS and without any of the exclusion criteria were candidates for the CMR examination, and these patients underwent TTE and CMR, within a 1-month period at Seoul National University Hospital and Samsung Medical Center. All anthropometric measures, the functional status and the medication data were acquired on the day of enrollment.

### Echocardiography

All patients underwent a comprehensive echocardiographic examination by experienced operators in accordance with the guideline of the European Association of Echocardiography [12]. The M-mode, 2-dimensional images, and Doppler recordings were obtained using adequate equipment (Vivid 7, GE Medical System, Horten, Norway). Measurements of left ventricular end-diastolic diameter, thickness of the interventricular septum and the posterior wall were made at end-diastole, which was defined as the frame after mitral valve closure or the frame in the cardiac cycle in which the cardiac dimension is the largest. The aortic valve area (AVA) was calculated with the continuity equation using the time velocity integral at the aortic valve and left ventricular outflow tract level. Transaortic mean PG was measured by using multiple transducer positions, i.e. apical 5 or 3 chamber, subcostal, right parasternal and suprasternal notch view. Each measurement was averaged for three cardiac cycles for patients in sinus rhythm and five cardiac cycles in atrial fibrillation. Global LV hemodynamic load was estimated using valvuloarterial impedance (*Z*_VA_), calculated as (systolic arterial pressure + transaortic mean gradient)/stroke volume index. Stroke volume index, measured by Doppler echocardiography with the continuity equation, was used to calculate Z_VA_ [13]. Body surface area (BSA) was calculated using the Mosteller formula and the AVA was divided by the BSA to calculate indexed AVA. All echocardiograms were interpreted by one cardiologist unaware of the purpose of the study, the patient’s condition and treatment, and blinded to the CMR measurements. In addition, all echocardiograms were cross-checked by the investigators after construction of the database and before starting the statistical analysis. Severe AS was defined as an AVA of less than 1.0cm^2^ plus either a transaortic mean PG of at least 40mmHg or a maximal transaortic velocity of at least 4.0m/sec [14].

### Cardiac magnetic resonance

The CMR images were taken using a standard 3.0-T scanner equipped with 6-channel phased-array receiver coils (Trio, Siemens, Erlangen, Germany). To quantify the LV function and mass, steady-state free precession cine images were acquired under an adequate breath-hold. The entire set of LV short-axis images was acquired from the base to apex so as to include the whole LV volume and these images were used for analysis of the LV volume, mass and systolic function. The imaging parameters were: echo time 1.6 msec, repetition time 3.6 msec, flip angle 80**°**, matrix size 256×150, slice thickness 6 mm with 4 mm gap between adjacent slices, FOV 240×300 cm, and temporal resolution 32 msec. The LV mass was measured with commercially available software (CMR42, Circle Cardiovascular Imaging Inc., Calgary, Canada). When quantifying the LV mass, the trabeculations and the papillary muscles were excluded from the LV mass, and the short axis stack was taken before contrast administration. The LV remodeling index (LVRI) was calculated by dividing the LV mass by the left ventricular end-diastolic volume [15, 16]. Since changes in LV geometry are dynamic, affecting both mass and volume, combining both parameters in a single index provides comprehensive information of the LV remodeling pattern due to chronic pressure overload [15]. Lastly, all of the CMR were performed consistent with the late gadolinium enhancement (LGE) protocols, as previously described [17]. Briefly, LGE images for myocardial fibrosis detection were obtained 10 minutes after injection of intravenous gadolinium (0.1 mmol/kg Magnevist; Schering, Berlin, Germany) followed by a flush of 20mL of saline at the same rate, using the phase-sensitive inversion recovery sequence. The most appropriate inversion time was set to null normal myocardium, usually between 280 and 360 msec. The protocol for the LGE images were as follows; echo time 42 msec, repetition time 9.1 msec, flip angle 13**°**, slice thickness 8 mm, interslice gap 2 mm, in-plane resolution 1.4×1.9 mm. The presence of LGE was adjudicated by an independent radiologist blinded to the protocol of the current study and 5SD definition was used for LGE quantification.[17] All CMR measurements were performed by a single independent expert technician blinded to the purpose of the study and echocardiographic measurements.

### Statistical analysis

The primary hypothesis of the study was that there would be gender-specific differences in the LV hypertrophic or remodeling response associated with AS. The main dependent variables, which were parameters of LV hypertrophic or remodeling response, were LVMI and LVRI, as measured by CMR. The independent variables were indexed AV area, transaortic mean PG and valvuloarterial impedance (*Z*_VA_), all implying the severity of AS or global LV hemodynamic load. We confirmed the normal distribution of the continuous parameters with the Kolmogorov-Smirnov test and visual inspection of the symmetricity of the histogram. Categorical variables were presented as numbers (percentages) and compared using the χ^2^ test or the Fisher’s exact test. Normally distributed continuous variables were expressed as means and standard deviations, and analyzed using the independent sample t-test. To explore the linear association between the LV remodeling response parameters and the AS severity parameters, we performed univariate linear regression and the interaction between the main associations according to the gender was also evaluated with the incorporation of interaction term into the model.

Since recently published studies showed that the trabeculations and papillary muscle could account for a substantial proportion of the LV mass [18–20], we also measured total LV mass with incorporation of the trabeculations and papillary muscle, and the overall statistical analysis was repeated to confirm the robustness of the original results.

We determined the independent predictors of LVMI and LVRI with multiple linear regression analysis. The covariates used in multivariate analysis were selected if the difference between the two groups was significant (p-value<0.1) or if they had predictive values. The covariates incorporated into the final model were: age >70, male, BMI, height, hypertension, diabetes mellitus, hyperlipidemia, transaortic mean PG, aortic valve area index, valvuloarterial impedance, bicuspid aortic valve, and NYHA functional class≥3. All probability values were two-sided and a p-value<0.05 was considered statistically significant. The statistical package SAS, version 9.3 (SAS Institute Inc., Cary, NC, USA) was used for statistical analyses.

## Results

### Baseline clinical characteristics and echocardiography parameters

A total of 118 patients with moderate or severe AS were enrolled in this registry. Baseline characteristics are summarized in Table 1. In brief, 53.4% of the patients were male, and the mean age of the study population was 67.4 years old. Symptomatic patients in New York Heart Association functional class III or IV accounted for 11.0% of the study population. There were no differences between the two genders in the baseline clinical characteristics, except that males had a higher BSA, height, baseline creatinine, and higher proportion of current smokers, compared with females. Although BMI was numerically higher in females, there were no significant differences in BMI nor in the proportion of obese patients, obesity being defined as BMI>25 kg/m^2^. In addition, there was no difference in the blood pressure level, medication regimen for blood pressure control, and the number of anti-hypertensive medications between the two genders (Table 1).

**Table 1:** Baseline clinical characteristics of the study participants.

	Total (n = 118)	Male (n = 63, 53.4%)	Female (n = 55, 46.6%)	P value
Age (years)	67.4 ± 9.6	67.6 ± 9.7	67.2 ± 9.7	0.822
Systolic blood pressure (mmHg)	127.0 ± 17.3	126.8 ± 15.7	127.2 ± 19.1	0.887
Diastolic blood pressure (mmHg)	70.3 ± 11.5	70.1 ± 11.2	70.6 ± 11.9	0.814
Body surface area (m^2^)	1.7 ± 0.2	1.8 ± 0.1	1.6 ± 0.1	<0.001
Height (cm)	160.4 ± 9.0	166.7 ± 5.7	153.1 ± 6.1	<0.001
Body mass index (kg/m^2^)	24.1 ± 2.9	23.9 ± 2.7	24.4 ± 3.1	0.331
Obesity (BMI ≥25 kg/m^2^)	41 (34.7%)	19 (30.2%)	22 (40.0%)	0.263
Baseline creatinine (mg/dL)	0.90± 0.23	0.99 ± 0.19	0.80 ± 0.22	<0.001
Hypertension	63 (53.4%)	32 (54.2%)	31 (58.5%)	0.651
Medications				
- ACEI/ARB	42 (35.6%)	22 (34.9%)	20 (36.4%)	0.870
- Beta-blocker	35 (29.7%)	22 (34.9%)	13 (23.6%)	0.181
- Calcium channel blocker	27 (22.9%)	15 (23.8%)	12 (21.8%)	0.797
- Diuretics	26 (22.0%)	12 (19.0%)	14 (25.5%)	0.402
- Statin	41 (34.7%)	20 (31.7%)	21 (38.2%)	0.464
Total number of anti-HTN medication	1.1 ± 1.1	1.1 ± 1.1	1.1 ± 1.1	0.790
Diabetes mellitus	27 (22.9%)	12 (20.3%)	15 (28.3%)	0.325
Hyperlipidemia	22 (18.6%)	10 (16.9%)	12 (22.6%)	0.449
Current smoker	13 (11.0%)	12 (20.3%)	1 (1.9%)	0.002
Atrial fibrillation	8 (6.8%)	5 (8.5%)	3 (5.7%)	0.564
NYHA functional class≥3	13 (11.0%)	6 (10.2%)	7 (13.2%)	0.616

The data are presented as mean (SD) or number (percentage).

Abbreviations: ACEI/ARB, angiotensin converting enzyme inhibitor/angiotensin receptor blocker; BMI, body mass index; HTN, hypertension; NYHA, New York Heart Association.

The baseline echocardiography parameters are summarized in Table 2. Males showed larger LV end-diastolic diameter, interventricular septal and posterior wall thickness. The mean transaortic peak velocity was 4.7 m/s, the transaortic mean PG 54.0mmHg and the mean AVA 0.8 cm^2^. Although the AVA was larger in the male patients, this difference was nonsignificant when corrected with BSA (AVA index; 0.5±0.1cm^2^/m^2^ versus 0.5±0.1cm^2^/m^2^, respectively, p = 0.707). Of the patients, 81.4% were classified as severe AS. The global LV load did not differ between the two genders (Z_VA_, valvuloarterial impedance; 3.8±1.1mmHg·m^2^/mL versus 3.7±0.9mmHg·m^2^/mL, p = 0.555) nor was there a difference in the incidence of mild aortic insufficiency (41.3% versus 43.6%, p = 0.795) (Table 2).

**Table 2:** Echocardiographic and cardiovascular magnetic resonance (CMR) parameters of the study participants.

Echocardiographic parameters	Total (n = 118)	Male (n = 63, 53.4%)	Female (n = 55, 46.6%)	P value
LV end-diastolic diameter (mm)	49.5 ± 5.3	50.7 ± 5.6	48.1 ± 4.5	0.006
LV end-systolic diameter (mm)	30.5 ± 4.3	31.1 ± 4.7	29.8 ± 3.7	0.084
IVST (mm)	11.6 ± 4.3	12.4 ± 5.3	10.8 ± 2.4	0.041
PWT (mm)	10.9 ± 2.0	11.4 ± 1.9	10.4 ± 2.1	0.009
Aortic annulus diameter (mm)	20.7 ± 3.8	21.4 ± 3.9	19.9 ± 3.5	0.058
Impaired ejection fraction (< 50%) (%)	6 (5.1%)	4 (6.3%)	2 (3.6%)	0.503
Left atrium diameter by M-mode (mm)	42.9 ± 7.1	43.2 ± 6.8	42.5 ± 7.4	0.613
E/e’ (septal)	16.5 ± 7.4	14.5 ± 5.4	18.7 ± 8.7	0.004
Vmax (m/sec)	4.7 ± 0.8	4.6 ± 0.9	4.7 ± 0.8	0.516
AVA (cm^2^)	0.8 ± 0.2	0.8 ± 0.2	0.7 ± 0.2	0.006
AVA index (cm^2^/m^2^)	0.5 ± 0.1	0.5 ± 0.1	0.5 ± 0.1	0.707
Transaortic mean PG (mmHg)	54.0 ± 21.6	52.0 ± 19.9	56.2 ± 23.5	0.296
Z_VA_ (mmHg·m^2^/mL)	3.7 ± 1.0	3.8 ± 1.1	3.7 ± 0.9	0.555
Severe AS (%)	96 (81.4%)	47 (74.6%)	49 (89.1%)	0.044
Bicuspid AV (%)	16 (13.6%)	9 (14.3%)	7 (12.7%)	0.805
Combined mild AR (%)	50 (42.4%)	26 (41.3%)	24 (43.6%)	0.795
**CMR parameters**	**Total (n = 118)**	**Male (n = 63, 53.4%)**	**Female (n = 55, 46.6%)**	**P value**
LV end-diastolic volume index (mL/m^2^)	91.3 ± 24.0	94.3 ± 27.0	87.8 ± 19.7	0.131
LV end-systolic volume index (mL/m^2^)	32.5 ± 14.7	34.1 ± 15.3	30.7 ± 14.0	0.225
LV ejection fraction (%)	65.5 ± 9.2	64.8 ± 9.2	66.3 ± 9.2	0.394
LV mass (g)^†^	158.1 ± 55.9	179.9 ± 54.7	133.0 ± 46.2	<0.001
LV mass indexed by BSA (g/m^2^)	94.9 ± 30.2	102.6 ± 29.1	86.1 ± 29.2	0.003
LV remodeling index (g/mL)	1.1 ± 0.3	1.1 ± 0.2	1.0 ± 0.3	0.018
Presence of late gadolinium enhancement	46 (39.0%)	27 (42.9%)	19 (34.5%)	0.356
Late gadolinium enhancement (% of total LV)	2.6 ± 4.6	2.5 ± 4.1	2.7 ± 5.2	0.837

The data are presented as mean (SD), except adjusted mean (SE) in the body mass index adjusted LV mass.

^†^When quantifying the LV mass, the trabeculations and the papillary muscles were excluded.

Abbreviations: AR, aortic regurgitation; AS, aortic stenosis; AVA, aortic valve area; BMI, body mass index; BSA, body surface area; E, early diastolic velocity at the mitral valve tip; e’, early mitral annular velocity at the septal annulus; IVST, interventricular septal thickness; LV, left ventricle; PG, pressure gradient; PWT, posterior wall thickness; Vmax, maximal transaortic velocity; Z_VA_, valvuloarterial impedance.

### Comparison of gender-specific left ventricular hypertrophy and remodeling pattern using CMR

The baseline CMR parameters are summarized in Table 2. Although severe AS was marginally more common in females than males (74.6% versus 89.1%, p = 0.044), males showed significantly higher LV mass and LVMI (LV mass indexed with BSA), compared with females (LVMI; 102.6±29.1g/m^2^ versus 86.1±29.2g/m^2^, p = 0.003) (Table 2). This difference persisted even when the LV trabeculations and the papillary muscles were included within the LV mass (LVMI; 121.3±34.1g/m^2^ versus 103.2±31.1g/m^2^, p = 0.004) (S1 Table). In addition, the LVRI was significantly higher in male patients, compared with female patients (1.1±0.2g/mL versus 1.0±0.3g/mL, p = 0.018), and remained significantly higher in males even when the LV trabeculations and the papillary muscles were included within the LV mass (1.5±0.3g/mL versus 1.4±0.4g/mL, p = 0.025) (Table 2 and S1 Table). In contrast to the LVMI or LVRI, neither the proportion of patients with LGE (42.9% versus 34.5%, p = 0.356) nor the extent of LGE (2.5 ± 4.1% in male vs. 2.7 ± 5.2 in female, p = 0.837) differed between the two genders.

**Fig 1:**
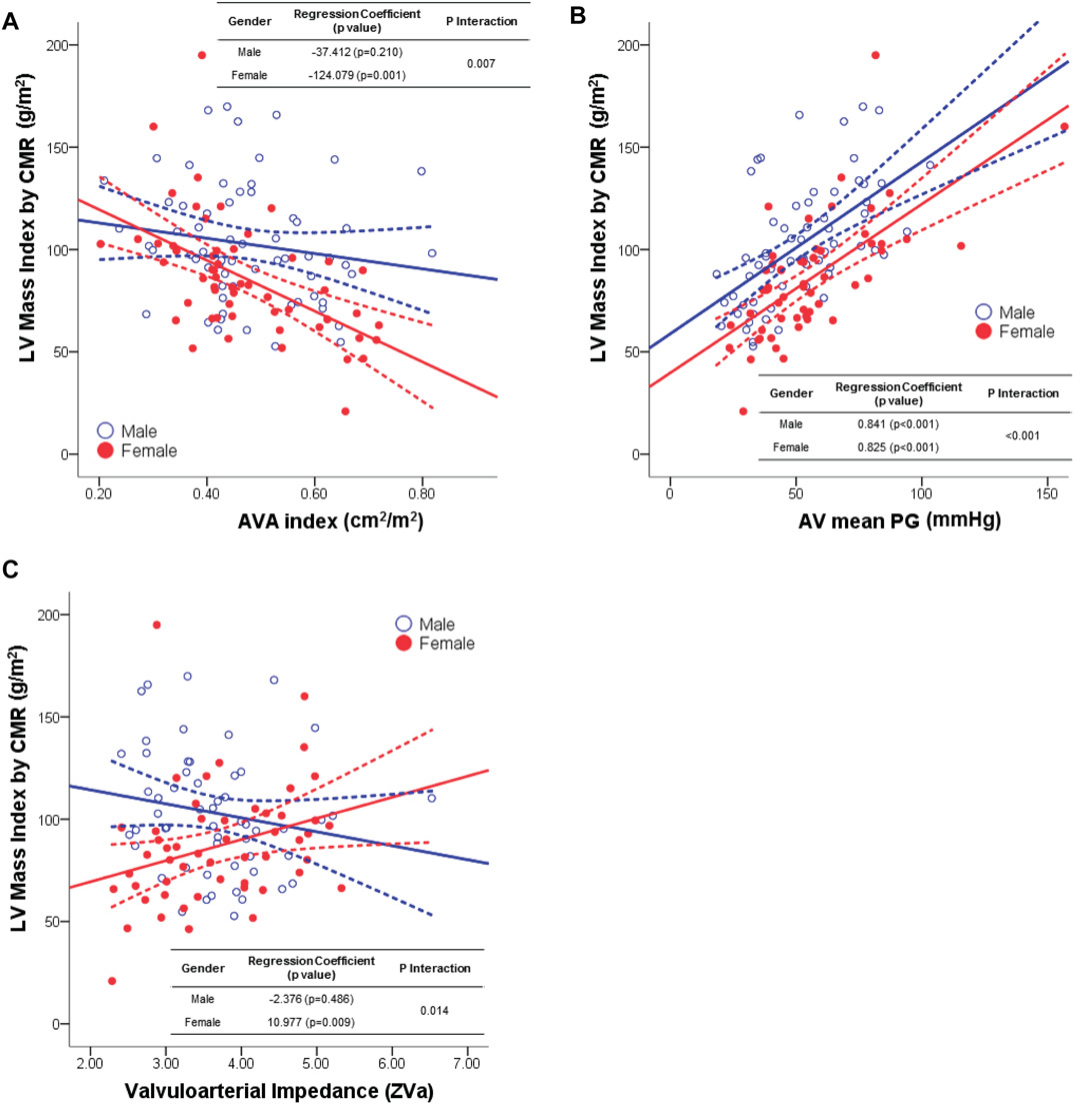
The association between left ventricular mass index and the severity of aortic stenosis or valvuloarterial impedance. Males consistently showed relatively higher left ventricular mass index in (A) larger aortic valve area index, (B) lower mean transaortic pressure gradient, or (C) lower valvuloarterial impedance, compared with females. However, there were significant differences between the two genders in the degree of correlation between the left ventricular mass index and the above three parameters. The univariate linear regression coefficient and the interaction p-value across the gender are shown. Abbreviations: AV, aortic valve; AVA, aortic valve area; CMR, cardiovascular magnetic resonance; PG, pressure gradient.

**Fig 2:**
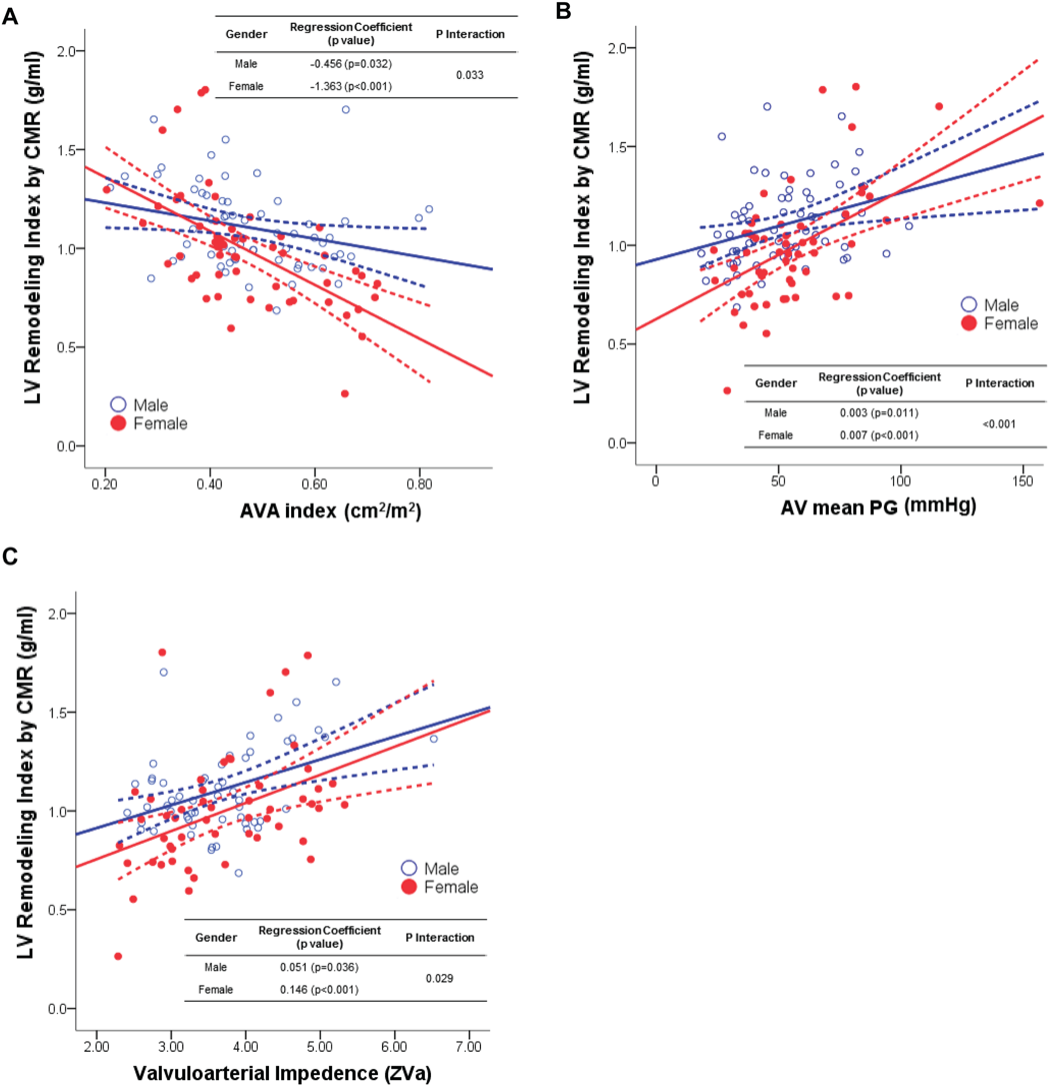
The association between left ventricular remodeling index and the severity of aortic stenosis or valvuloarterial impedance. Males consistently showed relatively higher left ventricular remodeling index in (A) larger aortic valve area index, (B) lower mean transaortic pressure gradient, or (C) lower valvuloarterial impedance, compared with females. However, there were significant differences between the two genders in the degree of correlation between the left ventricular remodeling index and the above three parameters. The univariate linear regression coefficient and the interaction p value across the gender are shown. Abbreviations: AV, aortic valve; AVA, aortic valve area; CMR, cardiovascular magnetic resonance; PG, pressure gradient.

In the scatter plot between the LVMI measured by CMR and the AS severity parameters, the LVMI closely correlated with the transaortic mean PG in both males and females. In addition, the LVRI also correlated with AVA index, AV mean PG and valvuloarterial impedance in both gender (Table 3). In a univariate linear regression analysis of the gender-specific interaction between the LVMI and parameters of AS severity, the LVMI showed significant interaction across gender with AVA index (P_Interaction_ = 0.007), transaortic mean PG (P_Interaction_<0.001) and valvuloarterial impedance (P_Interaction_ = 0.014) (Fig. 1 and Table 3). As with the LVMI, the LVRI also showed a significantly different association between males and females with the change of AVA index (P_Interaction_ = 0.033), transaortic mean PG (P_Interaction_<0.001) and valvuloarterial impedance (P_Interaction_ = 0.029) (Fig. 2 and Table 3). This significant interaction between the two genders tended to persist even when the trabeculations and the papillary muscles were included in the total LV mass (S2 Table, S1 Fig. and S2 Fig.). In addition, the gender-specific differences and interactions were maintained even after exclusion of the patients with moderate AS (Table 4).

**Table 3:** Univariate analysis of the association and gender-specific interaction between the indexed left ventricular mass or remodeling index and parameters of aortic stenosis severity.

Dependent Variable^†^	Independent Variable^‡^	Gender	Regression coefficient	CI	P value	R^2^	P_Interaction_
LV Mass Index	AVA index (cm^2^/m^2^)	Male	-37.412	-96.438, 21.613	0.210	0.026	0.007
		Female	-124.079	-178.59, -69.56	<0.001	0.282	
	AV mean PG (mmHg)	Male	0.841	0.533, 1.149	<0.001	0.328	<0.001
		Female	0.825	0.568, 1.081	<0.001	0.440	
	Z_VA_ (mmHg/mL/m^2^)	Male	-2.376	-9.151, 4.399	0.486	0.008	0.014
		Female	10.977	2.832, 19.121	0.009	0.121	
LV Remodeling Index	AVA index (cm^2^/m^2^)	Male	-0.456	-0.871, -0.042	0.032	0.074	0.033
		Female	-1.363	-1.882, -0.843	<0.001	0.343	
	AV mean PG (mmHg)	Male	0.003	0.001, 0.006	0.011	0.102	<0.001
		Female	0.007	0.004, 0.009	<0.001	0.276	
	Z_VA_ (mmHg/mL/m^2^)	Male	0.051	0.003, 0.098	0.036	0.070	0.029
		Female	0.146	0.070, 0.223	<0.001	0.217	

^†^Calculated from measurements using CMR.

^‡^AVA index and AV mean PG were measured by TTE.

Abbreviations: AV, aortic valve; AVA, aortic valve area; CMR, cardiovascular magnetic resonance; CI, confidence interval; LV, left ventricle; PG, pressure gradient; TTE, transthoracic echocardiography; Z_VA_, valvuloarterial impedance.

**Table 4:** Univariate analysis of the association and gender specific interaction between the indexed left ventricular mass or remodeling index and parameters of aortic stenosis severity, with exclusion of the patients with moderate AS.

Dependent Variable^†^	Independent Variable^‡^	Gender	Regression coefficient	CI	P value	R^2^	P_Interaction_
LV Mass Index	AVA index (cm^2^/m^2^)	Male	-30.124	-132.88, 72.629	0.558	0.008	<0.001
		Female	-122.867	-193.03, -52.710	0.001	0.209	
	AV mean PG (mmHg)	Male	0.964	0.547, 1.380	<0.001	0.325	0.036
		Female	0.758	0.481, 1.035	<0.001	0.392	
	Z_VA_ (mmHg/mL/m^2^)	Male	-3.084	-10.550, 4.381	0.410	0.015	0.152
		Female	8.376	-0.159, 16.912	0.054	0.077	
LV Remodeling Index	AVA index (cm^2^/m^2^)	Male	-0.988	-1.617, -0.360	0.003	0.182	<0.001
		Female	-1.435	-2.098, -0.772	<0.001	0.288	
	AV mean PG (mmHg)	Male	0.004	0.001, 0.007	0.016	0.123	0.039
		Female	0.006	0.003, 0.009	0.001	0.220	
	Z_VA_ (mmHg/mL/m^2^)	Male	0.056	0.008, 0.104	0.023	0.109	0.277
		Female	0.122	0.041, 0.203	0.004	0.164	

^†^Calculated from measurements using CMR.

^‡^AVA index and AV mean PG were measured by TTE.

Abbreviations: AV, aortic valve; AVA, aortic valve area; CMR, cardiovascular magnetic resonance; CI, confidence interval; LV, left ventricle; PG, pressure gradient; TTE, transthoracic echocardiography; Z_VA_, valvuloarterial impedance.

### Independent predictors of left ventricular mass index and remodeling index

In the multiple linear regression analysis to explore the independent predictors of the LVMI, male gender was significantly associated with increased LVMI, being 22.303 g/m^2^ (95% CI 8.063–36.543, p = 0.002) higher in males compared to females. Also, there was an apparent trend towards increased LVMI with increasing transaortic mean PG (regression coefficient 0.986, 95% CI 0.682–1.291, p<0.001). As with the LVMI, LVRI was also significantly increased with male gender (regression coefficient 0.150, 95% CI 0.018–0.281, p = 0.026), transaortic mean PG (regression coefficient 0.005, 95% CI 0.002–0.008, p<0.001) and the valvuloarterial impedance (Z_VA_; regression coefficient 0.076, 95% CI 0.009–0.144, p = 0.026) (Table 5). These findings were consistent even when the trabeculations and the papillary muscles were included in the total LV mass (S3 Table). Lastly, the multivariate model with inclusion of only the patients with severe AS showed that the independent predictors for LVMI or LVRI were male gender and transaortic mean PG. (Table 6).

**Table 5:** Determinants of left ventricular mass index or left ventricular remodeling index.^†^

Variables	Left Ventricular Mass Index			Left Ventricular Remodeling Index		
	Regression coefficient	95% CI	P value	Regression coefficient	95% CI	P value
Age > 70 years	-9.771	-19.708–0.165	0.054	0.015	-0.077–0.107	0.743
Male	22.303	8.063–36.543	0.002	0.150	0.018–0.281	0.026
Hypertension	5.4567	-4.474–15.409	0.278	0.029	-0.062–0.121	0.525
Diabetes Mellitus	2.133	-9.157–13.423	0.709	0.017	-0.087–0.121	0.746
AVA index	28.716	-41.61–99.04	0.958	0.089	-0.560–0.738	0.786
AV mean PG	0.986	0.682–1.291	<0.001	0.005	0.002–0.008	<0.001
Z_VA_	2.546	-4.729–9.820	0.489	0.076	0.009–0.144	0.026

^†^Evaluated by multiple linear regression model. Included covariates were age more than 70, male, body mass index (BMI), height, hypertension, diabetes mellitus, hyperlipidemia, transaortic mean pressure gradient, aortic valve area index, valvuloarterial impedance, bicuspid aortic valve, and NYHA functional class ≥3.

**Table 6:** Determinants of left ventricular mass index or left ventricular remodeling index with exclusion of the patients with moderate AS.^†^

Variables	Left Ventricular Mass Index			Left Ventricular Remodeling Index		
	Regression coefficient	95% CI	P value	Regression coefficient	95% CI	P value
Age > 70 years	-9.598	-20.386, 1.191	0.080	-0.002	-0.097, 0.094	0.970
**Male**	**16.562**	**4.973, 28.151**	**0.006**	**0.113**	**0.010, 0.216**	**0.031**
Hypertension	7.040	-3.974, 18.053	0.207	0.032	-0.066, 0.129	0.520
Diabetes Mellitus	0.293	-12.801, 13.386	0.965	-0.001	-0.117, 0.115	0.987
AVA index	-27.131	-124.50, 70.234	0.581	-0.628	-1.491, 0.235	0.152
**AV mean PG**	**0.852**	**0.522, 1.182**	**<0.001**	**0.003**	**0.001, 0.006**	**0.020**
Z_VA_	0.517	-8.060, 9.095	0.905	0.041	-0.035, 0.117	0.289

^†^Evaluated by multiple linear regression model. Included covariates were age more than 70, male, body mass index (BMI), height, hypertension, diabetes mellitus, hyperlipidemia, transaortic mean pressure gradient, aortic valve area index, valvuloarterial impedance, bicuspid aortic valve, and NYHA functional class ≥3.

## Discussion

This is one of the first reports to use CMR to investigate both the gender-specific difference of the hypertrophic and remodeling response and the interaction of these parameters with the hemodynamic parameters observed in AS patients. We demonstrated the gender-specific differences in the association between AS severity and the LVMI or LVRI.

More specifically, the LVMI and LVRI were significantly associated with transaortic mean PG, indexed AVA, and valvuloarterial impedance in both genders (except for the nonsignificant association between indexed AVA/valvuloarterial impedance and LVMI in males). More importantly, there was a significant interaction between genders and the regression coefficients, suggesting that the LV remodeling process differs between the two genders. In addition, independent predictors of LVMI were male gender and transaortic mean PG, whereas significant predictors of LVRI were male gender, transaortic mean PG and valvuloarterial impedance, even after adjusting with baseline characteristics including hypertension, height, and BMI. The incorporation of the LV trabeculations and papillary muscle into the total LV mass did not alter the overall results regarding the gender-specific differences in LV hypertrophy and remodeling patterns. To summarize, although males are associated with more LVH and a higher LV remodeling index even with a similar degree of AS severity or global LV hemodynamic load, females showed a more exaggerated LV remodeling response with increased severity of AS and hemodynamic loads.

Previously, Milavetz et al. pointed out that the differences in LV geometry between males and females were largely eliminated after normalizing for BSA [3]. Since all of the studies reporting gender-specific differences in the LV geometry and the pattern of LVH used TTE-measured values and the calculation of LV mass incorporates the cavity dimension, all of which are usually higher in men, the disappearance of sex differences after adjustment with BSA may not be an unexpected result [9, 10]. Although TTE is still a standard and most commonly used method for assessment of LVM in clinical practice, LVM calculation based on TTE measurements have been known to overestimate it, especially in patients with LVH [21]. Notably, our study demonstrated gender-specific differences in the LVH and remodeling with the use of CMR, which has been the “gold-standard” for assessment of LVM and volume in recent studies. Previous studies have demonstrated no significant difference in the progression of AS between males and females, and gender was not an independent predictor of AS progression [22–24]. Therefore, the noted difference in LV remodeling pattern, presented as LVMI and LVRI, is less likely to be influenced neither by any gender difference in the disease duration, nor the rate of AS progression. Rather, the differences in LV remodeling pattern between males and females are more likely to arise from the difference in gender itself.

Our analysis showing the gender-specific differences in LVH and remodeling might also be an important possible mechanism for gender-specific differences in the prognostic profile after definite treatment of AS (i.e. SAVR or TAVR) [6, 7]. Although there have been debates about the gender-specific differences in the post-operative prognosis after SAVR [4, 5], recently published subgroup analysis of the PARTNER 1A trial [8] and the results of a registry-based study [6, 7] have shown better short- and mid-term survival in female patients, in up to 2 years of follow-up. Considering diverse confounding factors which could impact the outcome after SAVR (for example, the technical difficulty of surgical AVR in females due to smaller stature, BSA, aortic root, and subsequent prosthesis-patient mismatch, etc), clinical outcomes after TAVR might be more directly influenced by the improvement of pressure overload itself [6–8], which is supported by recent reports as listed above.

Recently, Dweck et al. characterized the pattern of LVH and remodeling with 91 patients with moderate to severe AS, using CMR [16]. They reported that the only determinant of LVMI was male sex (regression coefficient 13.8 g/m^2^, 95% CI 2.8–24.7, p = 0.02); however, AVA or AVA index was not significantly associated with LVMI. In addition, the lack of correlation between AVA and LVMI persisted in sub-group analyses of gender (male: r^2^ = 0.000, p = 0.91; female: r^2^ = 0.020, p = 0.44). Nonetheless, Dweck et al. only used AVA as parameters of AS severity, and did not evaluate the relationship between transaortic mean PG and LVMI. In our results, male gender was an independent determinant of LVMI (regression coefficient 17.42 g/m^2^, 95% CI 1.49–33.35, p = 0.033), but not AVA index, in agreement with the report of Dweck et al. By contrast, although AVA index was not significantly associated with LVMI in the overall population, the association was significant in the female subgroup, therefore there was significant interaction across gender and the association between AVA index and LVMI. In addition, transaortic mean PG was another independent determinant of LVMI in both genders, and this linear association was different across genders, with a significant interaction p-value. Although the exact mechanism to explain the correlation between AS severity parameter and LV hypertrophy or remodeling index was unclear, a previous pre-clinical study which demonstrated that females undergo a faster regression of LVH after unloading of the LV pressure suggests that there might be at least some correlation between any parameter of AS degree and the degree of LVH [25]. The differences between the results of Dweck et al. and ours can be partially explained by the following aspects; First of all, the number of patients enrolled was larger in the current study, especially female subjects. Second, the degree of AS severity was also different. The mean AVA was 0.93cm^2^ in the previous study, whereas it was 0.8cm^2^ in our study, which is also reflected by the Vmax as well (3.4~4.0m/sec (The mean value of the total population is not provided.) vs. 4.7m/sec). Lastly, we think it would be more reasonable to draw the correlation between AVA index and the LV mass index (as in ours) because the AVA may vary between patients with extreme habitus. The correlation drawn in the previous paper was shown between the AVA and the LV mass index, which leaves out the importance of body size into account when considering the AS severity.

It should be noticed that there may be possible influence of differences in the baseline LV mass between the two genders. For example, the difference in the LV mass of normal male and female volunteers are significantly different across the different ethnicities (93 vs. 65, mass difference between two genders 28 g in the normal Korean volunteers (unpublished data); 128 vs. 87, mass difference between two genders 41 g in the normal Western volunteers [26]). However, it would be hard to suggest that the difference in the LVH between the two genders is wholly attributed to the baseline difference in the LV mass before AS ensues because the difference of LV mass between the genders clearly shows significant difference as compared to our preliminary data in normal volunteers (180 vs. 133, mass difference between two genders 47 g in our data from AS patients). Likewise, we think our data shows a clear difference between males and females in the response of the LV to adapt to the pressure overload.

Our study is not without limitations. First, this was a cross-sectional study using CMR in moderate or severe AS patients. Therefore, serial changes in LVH and remodeling after treatment could not be evaluated, the results of which we anticipate in the future. Second, we focused on the assessment of LVH and remodeling in response to chronic pressure overload, but could not provide clinical outcomes after treatment of AS and therefore, could not correlate this with prognostic findings. Therefore our findings—of the significantly higher development of LVH and remodeling in male patients—should be considered as hypothesis-generating for future treatment-guided longitudinal trials. Multicenter longitudinal trials are warranted to clarify the prognostic consequences of different LV responses across gender following treatment of AS. Third, over one half of patients had co-existing hypertension, which may be another major factor in LV hypertrophy and remodeling. The duration of hypertension could not be obtained from each patient since the participating two centers are highest tertiary referral centers in Korea and almost all patients with AS had been referred from primary or secondary medical centers. Although the prevalence or blood pressure level of and medication pattern including total number of anti-hypertensive medications did not differ between males and females, the possible effect of hypertension on the LVH and remodeling cannot be fully eliminated. However, considering that hypertension and AS commonly co-exist, it was not desirable to exclude all hypertensive patients, as this would affect the ability of the results to be generalized. Lastly, the proportion of the patients with obesity was substantially lower in our population and it has been shown that obesity itself can influence the LV remodeling [26]. Therefore, some ethnic difference should be taken into account when interpreting the current analysis.

## Conclusions

In conclusion, we have demonstrated gender-specific differences in the pattern of LV response in moderate to severe AS patients. The LVMI and LVRI were significantly associated with AS severity indices, with different patterns according to gender. Independent predictors of LVMI or LVRI were male gender and transaortic mean PG, even with the adjustment for hypertension. Our data suggest a significant increase in LV mass and consequently further progression in degree of LV remodeling in males, however, females show a more exaggerated LV remodeling response following the increased severity of AS and hemodynamic loads, compared with males. The relationship of these findings with clinical outcomes warrants further investigation.

## Supporting Information

S1 Table Cardiovascular magnetic resonance (CMR) parameters with inclusion of trabeculations and the papillary muscles into the LV mass. (DOC)

S2 Table Univariate analysis of the association and gender-specific interaction between the indexed left ventricular mass or remodeling index and parameters of aortic stenosis severity, with the trabeculations and the papillary muscles included in the LV mass. (DOC)

S3 Table Determinants of left ventricular mass index or left ventricular remodeling index with the trabeculations and the papillary muscles included in the LV mass. (DOC)

S1 Fig The association between left ventricular mass index and the severity of aortic stenosis or valvuloarterial impedance, with the trabeculations and the papillary muscles included in the LV mass. Males consistently showed relatively higher left ventricular mass index in (A) larger aortic valve area index, (B) lower mean transaortic pressure gradient, or (C) lower valvuloarterial impedance, compared with females. However, there were significant differences between the two genders in the degree of correlation between the left ventricular mass index and the above three parameters. The univariate linear regression coefficient and the interaction p-value across the gender are shown. Abbreviations: AV, aortic valve; AVA, aortic valve area; CMR, cardiovascular magnetic resonance; LVMI, left ventricular mass index; PG, pressure gradient.
(TIF)

S2 Fig The association between left ventricular remodeling index and the severity of aortic stenosis or valvuloarterial impedance, with the trabeculations and the papillary muscles included in the LV mass. Males consistently showed relatively higher left ventricular remodeling index in (A) larger aortic valve area index, (B) lower mean transaortic pressure gradient, or (C) lower valvuloarterial impedance, compared with females. However, there were significant differences between the two genders in the degree of correlation between the left ventricular remodeling index and the above three parameters. The univariate linear regression coefficient and the interaction p value across the gender are shown. Abbreviations: AV, aortic valve; AVA, aortic valve area; CMR, cardiovascular magnetic resonance; PG, pressure gradient.
(TIF)
