# Supplementary material for: Gender Difference in Ventricular Response to Aortic Stenosis: Insight from Cardiovascular Magnetic Resonance
Source: PLoS One. 2015 Mar 26;10(3):e0121684. doi: 10.1371/journal.pone.0121684 (PMC4374835; doi:10.1371/journal.pone.0121684)
Supplement: S1 Table — (DOC) [file pone.0121684.s001.doc]

**S1 Table.** Cardiovascular magnetic resonance (CMR) parameters with inclusion of trabeculations and the papillary muscles into the LV mass.^†^

| **CMR parameters** | **Total (n=96)** | **Male (n=47, 49.0%)** | **Female (n=49, 51.0%)** | **P value** |
| --- | --- | --- | --- | --- |
| LV mass (g) | 187.9 ± 63.9 | 212.7 ± 64.7 | 160.0 ± 50.4 | <0.001 |
| LV mass indexed by BSA (g/m^2^) | 112.8 ± 33.8 | 121.3 ± 34.1 | 103.2 ± 31.1 | 0.004 |
| Papillary muscle mass (g) | 28.7 ± 11.4 | 31.4 ± 12.4 | 25.5 ± 9.3 | 0.005 |
| Proportion of papillary muscle (%) | 15.4 ± 3.1 | 14.7 ± 2.7 | 16.2 ± 3.4 | 0.012 |
| LV end-diastolic volume index (mL/m^2^) | 78.3 ± 20.2 | 81.7 ± 22.2 | 74.4 ± 16.9 | 0.047 |
| LV remodeling index (g/mL) | 1.5 ± 0.3 | 1.5 ± 0.3 | 1.4 ± 0.4 | 0.025 |

^†^ The data are presented as mean (SD). Abbreviations: BSA, body surface area; LV, left ventricle.
