# Supplementary material for: Gender Difference in Ventricular Response to Aortic Stenosis: Insight from Cardiovascular Magnetic Resonance
Source: PLoS One. 2015 Mar 26;10(3):e0121684. doi: 10.1371/journal.pone.0121684 (PMC4374835; doi:10.1371/journal.pone.0121684)
Supplement: S2 Table — (DOC) [file pone.0121684.s002.doc]

**S2 Table.** Univariate analysis of the association and gender specific interaction between the indexed left ventricular mass or remodeling index and parameters of aortic stenosis severity, with the trabeculations and the papillary muscles included in the LV mass.

| **Dependent Variable**^†^ | **Independent Variable**^‡^ | **Gender** | **Regression coefficient** | **CI** | **P value** | **R^2^** | **P_Interaction_** |
| --- | --- | --- | --- | --- | --- | --- | --- |
| LV Mass Index | AVA index (cm^2^/m^2^) | Male | -30.321 | -100.92, 40.279 | 0.394 | 0.012 | <0.001 |
|  |  | Female | -127.265 | -186.08, -68.454 | <0.001 | 0.262 |  |
|  | AV mean PG (mmHg) | Male | 0.936 | 0.565, 1.307 | <0.001 | 0.298 | 0.013 |
|  |  | Female | 0.849 | 0.569, 1.128 | <0.001 | 0.411 |  |
|  | Z_VA_ (mmHg/mL/m^2^) | Male | -3.838 | -11.751, 4.074 | 0.336 | 0.015 | 0.015 |
|  |  | Female | 11.009 | 2.276, 19.741 | 0.014 | 0.108 |  |
| LV Remodeling Index | AVA index (cm^2^/m^2^) | Male | -0.625 | -1.214, -0.036 | 0.038 | 0.070 | <0.001 |
|  |  | Female | -1.647 | -2.294, -1.000 | <0.001 | 0.330 |  |
|  | AV mean PG (mmHg) | Male | 0.004 | 0.001, 0.008 | 0.018 | 0.090 | 0.027 |
|  |  | Female | 0.007 | 0.003, 0.011 | <0.001 | 0.222 |  |
|  | Z_VA_ (mmHg/mL/m^2^) | Male | 0.069 | 0.003, 0.135 | 0.041 | 0.068 | 0.046 |
|  |  | Female | 0.183 | 0.089, 0.277 | <0.001 | 0.224 |  |

^†^Calculated from measurements using CMR.

^‡^AVA index and AV mean PG were measured by TTE.

Abbreviations: AV, aortic valve; AVA, aortic valve area; CMR, cardiovascular magnetic resonance; CI, confidence interval; LV, left ventricle; PG, pressure gradient; TTE, transthoracic echocardiography; Z_VA_, valvuloarterial impedance.
