# Supplementary material for: Gender Difference in Ventricular Response to Aortic Stenosis: Insight from Cardiovascular Magnetic Resonance
Source: PLoS One. 2015 Mar 26;10(3):e0121684. doi: 10.1371/journal.pone.0121684 (PMC4374835; doi:10.1371/journal.pone.0121684)
Supplement: S3 Table — (DOC) [file pone.0121684.s003.doc]

**S3 Table.** Determinants of left ventricular mass index or left ventricular remodeling index with the trabeculations and the papillary muscles included in the LV mass.

| **Variables** | **Left Ventricular Mass Index** | | | **Left Ventricular Remodeling Index** | | |
| --- | --- | --- | --- | --- | --- | --- |
|  | **Regression coefficient** | **95% CI** | **P value** | **Regression coefficient** | **95% CI** | **P value** |
| Age > 70 years | -12.328 | -23.741, -0.916 | 0.035 | 0.020 | -0.101, 0.142 | 0.738 |
| Male | 23.254 | 6.928, 39.580 | 0.006 | 0.173 | 0.001, 0.346 | 0.048 |
| Hypertension | 4.453 | -6.963, 15.868 | 0.441 | 0.013 | -0.108, 0.135 | 0.827 |
| Diabetes Mellitus | 3.442 | -9.532, 16.416 | 0.600 | 0.041 | -0.097, 0.178 | 0.561 |
| AVA index | 38.304 | -43.017, 119.624 | 0.352 | 0.033 | -0.831, 0.897 | 0.940 |
| AV mean PG | 1.073 | 0.724, 1.422 | <0.001 | 0.006 | 0.002, 0.009 | 0.003 |
| Z_VA_ | 2.319 | -6.049, 10.687 | 0.584 | 0.090 | 0.001, 0.179 | 0.047 |

^†^Evaluated by multiple linear regression model. Included covariates were age more than 70, male, body mass index (BMI), height, hypertension, diabetes mellitus, hyperlipidemia, transaortic mean pressure gradient, aortic valve area index, valvuloarterial impedance, bicuspid aortic valve, and NYHA functional class ≥3.
