# Supplementary figures and images for: Gender Difference in Ventricular Response to Aortic Stenosis: Insight from Cardiovascular Magnetic Resonance
Source: PLoS One. 2015 Mar 26;10(3):e0121684. doi: 10.1371/journal.pone.0121684 (PMC4374835; doi:10.1371/journal.pone.0121684)

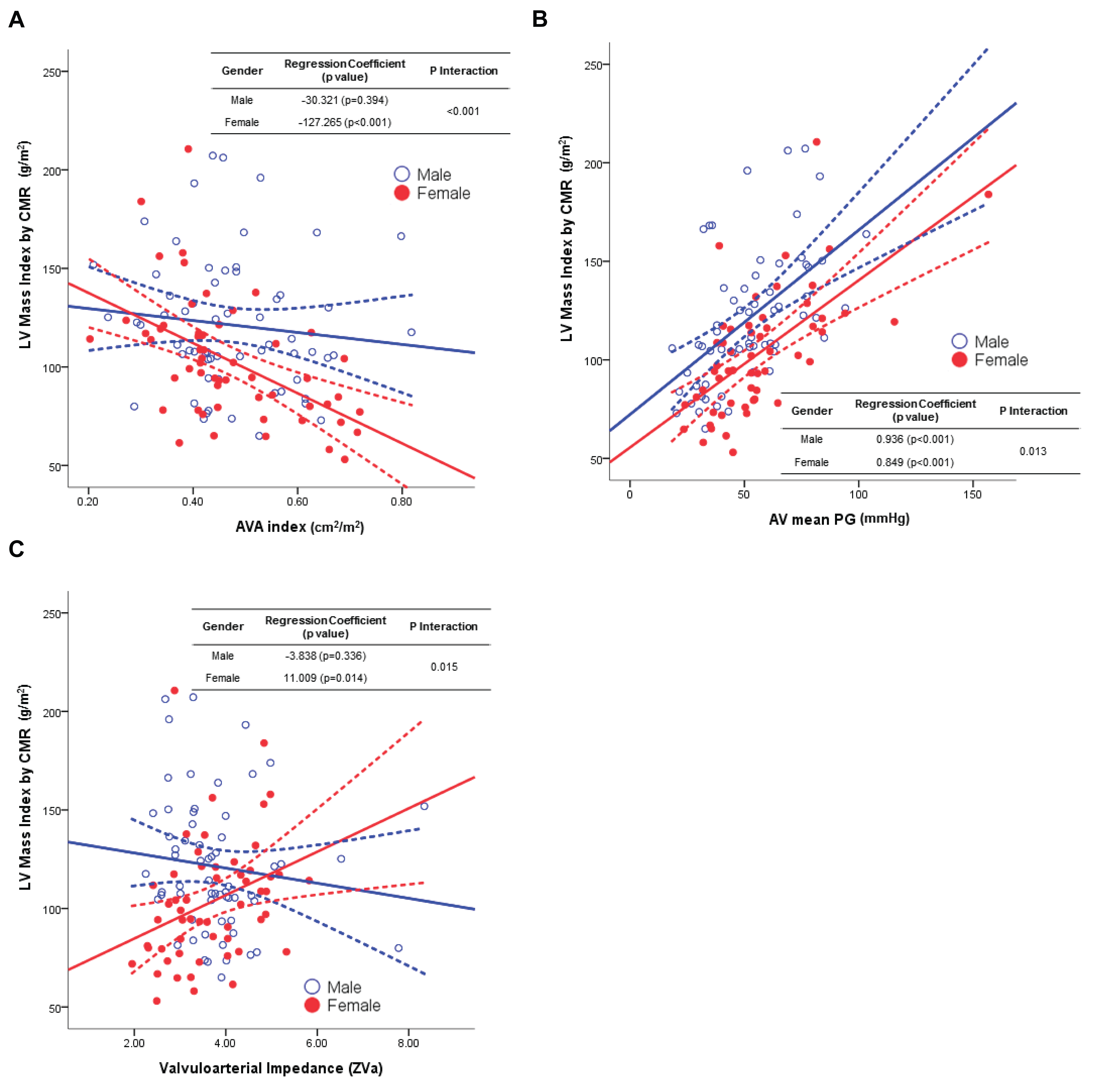

Supplement: S1 Fig — Males consistently showed relatively higher left ventricular mass index in (A) larger aortic valve area index, (B) lower mean transaortic pressure gradient, or (C) lower valvuloarterial impedance, compared with females. However, there were significant differences between the two genders in the degree of correlation between the left ventricular mass index and the above three parameters. The univariate linear regression coefficient and the interaction p-value across the gender are shown. Abbreviations: AV, aortic valve; AVA, aortic valve area; CMR, cardiovascular magnetic resonance; LVMI, left ventricular mass index; PG, pressure gradient. (TIF) [file pone.0121684.s004.tif]

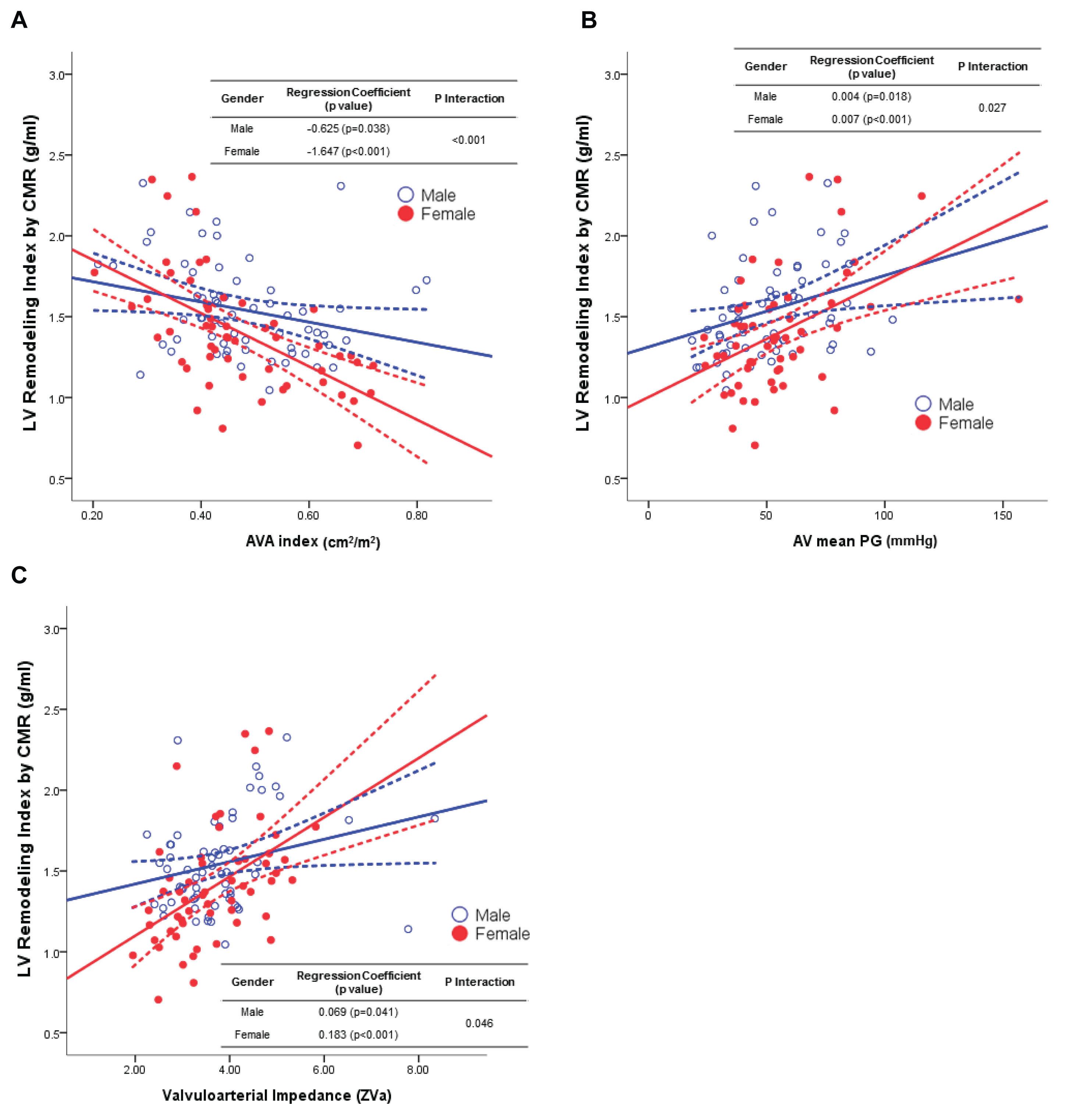

Supplement: S2 Fig — Males consistently showed relatively higher left ventricular remodeling index in (A) larger aortic valve area index, (B) lower mean transaortic pressure gradient, or (C) lower valvuloarterial impedance, compared with females. However, there were significant differences between the two genders in the degree of correlation between the left ventricular remodeling index and the above three parameters. The univariate linear regression coefficient and the interaction p value across the gender are shown. Abbreviations: AV, aortic valve; AVA, aortic valve area; CMR, cardiovascular magnetic resonance; PG, pressure gradient. (TIF) [file pone.0121684.s005.tif]
